# ChatGPT performance in answering medical residency questions in nephrology: a pilot study in Brazil

**DOI:** 10.1590/2175-8239-JBN-2024-0254en

**Published:** 2025-07-04

**Authors:** Helvécio Neves Feitosa, João Filipe Cavalcante Uchoa Furtado, Eduardo Correia Eulálio, Pedro Vianna Caldas Ribeiro, Lucas Macêdo Aurélio Paiva, Matheus Maia Gonçalves Bringel Correia, Geraldo Bezerra da Silva

**Affiliations:** 1 Universidade de Fortaleza, Faculdade de Medicina, Fortaleza, CE, Brazil.; 2 Universidade de Fortaleza, Faculdade de Medicina, Programas de Pós-Graduação em Saúde Pública e Ciências Médicas, Fortaleza, CE, Brazil.

**Keywords:** Generative Artificial Intelligence, Large Language Models, Medical Residency Exam, Nephrology

## Abstract

**Objective:**

This study evaluated the performance of ChatGPT 4 and 3.5 versions in answering nephrology questions from medical residency exams in Brazil.

**Methods:**

A total of 411 multiple-choice questions, with and without images, were analyzed, organized into four main themes: chronic kidney disease (CKD), hydroelectrolytic and acid-base disorders (HABD), tubulointerstitial diseases (TID), and glomerular diseases (GD). Questions with images were answered only by ChatGPT-4. Statistical analysis was performed using the chi-square test.

**Results:**

ChatGPT-4 achieved an overall accuracy of 79.80%, while ChatGPT-3.5 achieved 56.29%, with a statistically significant difference (p < 0.001). In the main themes, ChatGPT-4 performed better in HABD (79.11% vs. 55.17%), TID (88.23% vs. 52.23%), CKD (75.51% vs. 61.95%), and DG (79.31% vs. 55.29%), all with p < 0.001. ChatGPT-4 presented an accuracy of 81.49% in questions without images and 54.54% in questions with images, with an accuracy of 60% for electrocardiogram analysis. This study is limited by the small number of image-based questions and the use of outdated examination items, reducing its ability to assess visual diagnostic skills and current clinical relevance. Furthermore, addressing only 4 areas of Nephrology may not fully represent the breadth of nephrology practice.

**Conclusion:**

ChatGPT-3.5 was found to have limitations in nephrology reasoning compared to ChatGPT-4, evidencing gaps in knowledge. The study suggests that further exploration is needed in other nephrology themes to improve the use of these AI tools.

## Introduction

ChatGPT is an application based on large language models (LLMs), such as GPT-4, developed by OpenAI^1^. These models use complex neural networks to process and generate natural language in a fluent and coherent way^2^. The history of LLMs begins with advances in natural language processing (NLP), with a significant increase in the ability to generate text that resembles human writing. One example of this is ChatGPT, which has had a major impact since its launch in 2022^3^. Its training involves large volumes of data and techniques such as fine-tuning and reinforcement learning from human feedback^4^. This innovation has not only transformed research areas, but also revolutionized interactions with artificial intelligence (AI) in several disciplines^5^.

ChatGPT has been widely used to answer complex questions in medical residency exams, showing promising performance in several specialties^6,7,8^. For example, in a study that evaluated its ability to complete the Italian medical residency exam, the model achieved an overall accuracy of 90.44%, outperforming 99.6% of human participants6. In another study, ChatGPT achieved similar performance to plastic surgery residents, answering 55.8% of questions correctly^7^. In the field of nephrology, GPT was tested on self-assessment questions, achieving an accuracy of 54.5% with the GPT-4 version, which was significantly superior than previous versions, but still below that expected by experienced residents^8^.

The medical residency exam in Brazil is a required exam for medical graduates seeking specialized training. The exam is traditionally complete with multiple-choice and essay questions and is divided into broad areas such as General Surgery, Pediatrics, Obstetrics and Gynecology, Basic Sciences, Public Health, and Clinical Medicine^9^. This structured, general approach ensures that the candidate’s knowledge is assessed in a variety of medical fields, relevant to the desired specialization.

Understanding the differences in performance between GPT 3.5 and 4 is crucial, as each iteration of these models brings improvements that can be significant in clinical and educational settings. For example, the superior performance of GPT-4, as demonstrated in the study by Rosoł et al.^10^, suggests a greater approximation of human reasoning in complex medical scenarios, potentially providing a more reliable tool for medical training and decision-making support. It is worth noting that the importance of this research extends beyond academic curiosity. It focuses on the potential to support medical education with these models functioning as advanced tools for training and assessment, providing simulations and diagnostic aids to help medical students and professionals practice and refine their clinical reasoning in a risk-free environment. Furthermore, as AI models like GPT-4 continue to evolve, they offer new opportunities to enhance clinical reasoning by simulating real-world scenarios, enable continuous access to up-to-date medical knowledge, and facilitate individualized learning through tailored feedback, ultimately contributing to a more adaptive and effective medical education.

In this study, we compared the performance of GPT-4 and GPT-3.5 in answering multiple-choice questions from nephrology residency exams. Additionally, we assessed the models’ capabilities by analyzing the percentage of correct answers across various nephrology topics. To further evaluate the open-source models, we conducted a comparative analysis of common errors and performed subgroup evaluations based on question type, including those with and without visual components.

## Methods

### GPTs

In this study, we assessed the accuracy of GPT-4 and GPT-3.5 in answering multiple-choice nephrology questions. A prompt-following strategy was employed, where each prompt included “Context”, “Question”, and “Choice” to leverage the model’s large input token capacity, with one prompt per question^11^. If the model failed to provide a definitive answer, the response was marked as incorrect. The evaluations were performed using a local NVIDIA GPU to execute the commands.

### Inclusion Criteria

Only official medical residency selection exams aimed at general practitioners and administered for admission to programs in various subspecialties, not exclusively in Nephrology were selected. The questions analyzed corresponded to the Clinical Medicine section of these tests, actively obtained by the researchers from public digital repositories made available by the educational institutions themselves. There was no random selection: all questions related to Nephrology, with and without image interpretations, in these official exams were included to ensure representativeness of the content used in real selection processes. It is important to mention that the image-based questions, including those on electrocardiograms, were part of the exams intended for general practitioner candidates and not specifically designed for Nephrology specialists. Each question presented a clinical scenario, followed by a prompt to select the single correct answer from a list of options. The questions were not categorized by difficulty level, as the interpretation of such exams can vary greatly. What is considered challenging for one candidate might not be for another, depending on their academic background and prior experience.

### Exclusion Criteria

Questions related to Clinical Medicine that were not related to Nephrology were excluded. Questions involving patient scenarios with complex tables were excluded due to the challenges LLMs have in interpreting patient outcomes presented in table format. The 22 questions that required image interpretation were only included in GPT 4.0, given that only this version can read images.

### Dataset

**Figure 1:**
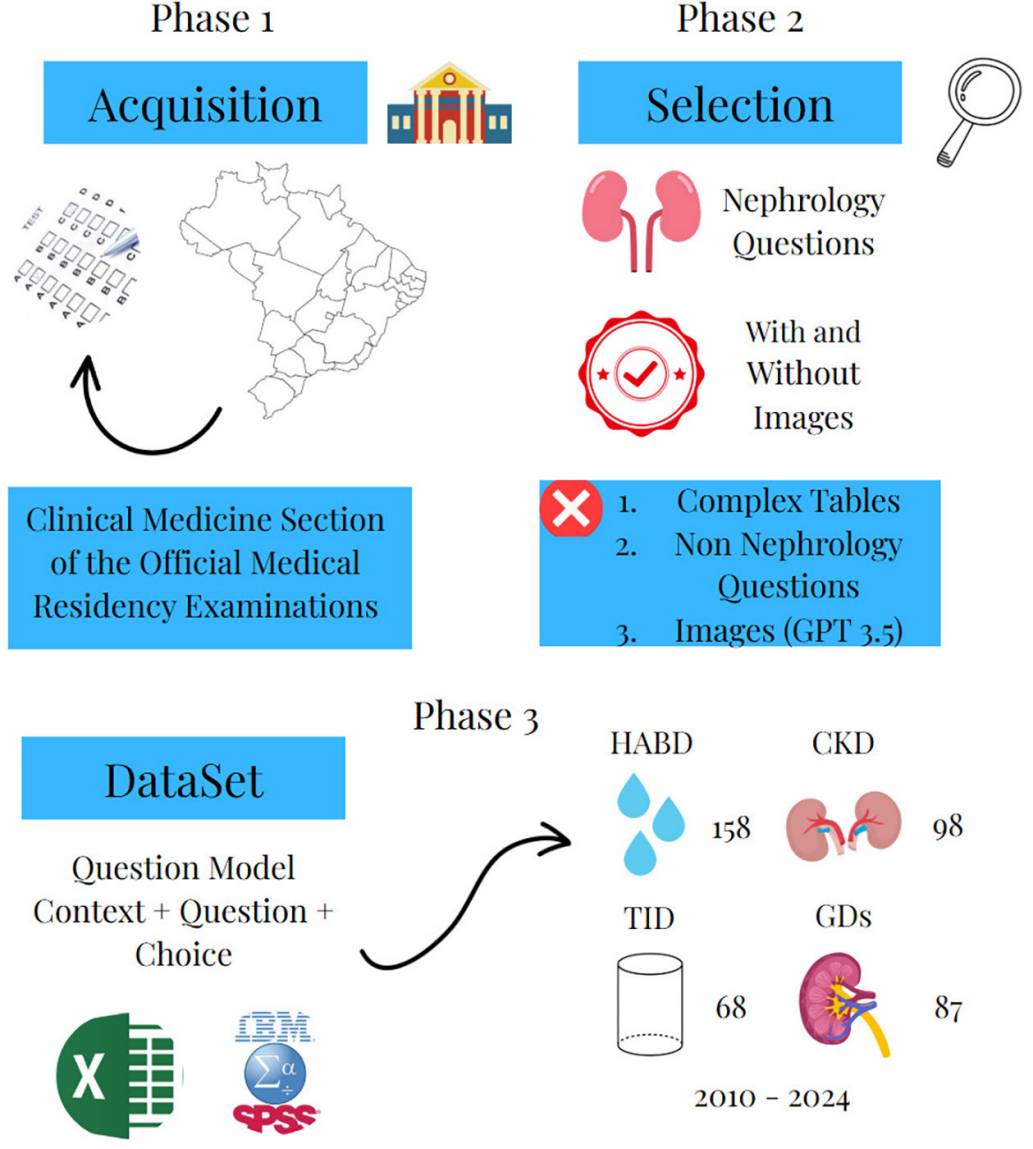
Process of collecting, selecting and analyzing the questions included.

After the question collection period, our dataset was composed of 411 multiple-choice questions extracted from 310 exams of 161 institutions divided into 4 main blocks: hydroelectrolytic and acid-base disorders (158 questions), tubulointerstitial diseases (68 questions), chronic kidney disease (98 questions), and glomerular diseases (87 questions). Questions were selected from medical residency exams conducted between January 2010 and March 2024, all administered in Brazilian Portuguese language. The questions were tested in September 2024. Of the 411 questions, 389 without images were selected for evaluation by both GPT-3.5 and GPT-4.0, while the 22 questions with images were evaluated exclusively by GPT-4.0. Figure 1 summarizes the process of question capture and selection as well as dataset creation.

### Experimental Analysis

To analyze the questions, we transferred the information into a Microsoft Excel spreadsheet to incorporate the responses acquired from the test bank. As a result, each example in our structured xlsx file included the question ID, context, prompt, answer options, correct answer, and the specific subject area of nephrology to which the question belonged. We used the Statistical Package for the Social Sciences (SPSS) version 28.0 to conduct the chi-square test and compare the performance of the two GPT models.

## Results

GPT-4 exhibited significantly higher overall performance compared to GPT-3.5, achieving an accuracy of 79.80% (328/411) versus 56.29% (219/389) for GPT-3.5, with this difference reaching statistical significance (p < 0.001; RR: 1.418, 95% CI: 1.282–1.567). When performance was stratified by nephrology subdomains, GPT-4 consistently outperformed GPT-3.5. In hydroelectrolytic and acid-base disorders (HABD), GPT-4 reached an accuracy of 79.11% (125/158) compared to 55.17% (80/145) for GPT-3.5 (RR: 1.434, 95% CI: 1.213–1.695, p < 0.001). For tubulointerstitial diseases (TID), the rates were 88.23% (60/68) versus 52.23% (35/67) for GPT-4 and GPT-3.5, respectively (RR: 1.689, 95% CI: 1.322–2.158, p < 0.001). In chronic kidney disease (CKD), GPT-4 achieved 75.51% (74/98), whereas GPT-3.5 reached 61.95% (57/92), with a relative risk of 1.219 (95% CI: 1.002–1.482, p < 0.001). In glomerular diseases (GDs), GPT-4 showed an accuracy of 79.31% (69/87) compared to 55.29% (47/85) for GPT-3.5 (RR: 1.434, 95% CI: 1.152–1.786, p < 0.001). Thus, across all evaluated domains, GPT-4 demonstrated a clearly and statistically significant better accuracy than GPT-3.5.

**Figure 2:**
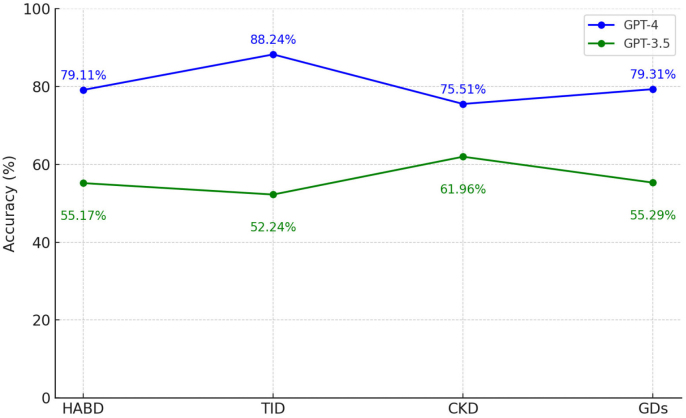
Line graph of GPT accuracy by area.

GPT-4 successfully answered 317 of 389 text-only queries and 12 of 22 image-based inquiries, corresponding 81.49% and 54.54%, respectively. The trajectory of correct answers in several question banks indicated an almost linear increase for GPT-4, unlike GPT-3.5, which was not comparable in any segmented assessment (Figure 2). Table 1 shows the summary of correct answers for LLMs in general and in the topics.

**Table 1:** Descriptive and comparative analysis of GPT performance

Performance	GPT 3.5, n (%)	GPT 4.0, n (%)	Questions with image, n (%)	RR (95%CI); (GPT 4 vs GPT 3.5)	p-value
Overall	219/389 (56.29%)	328/411 (79.80%)	12/22 (54.54%)	1.418 (1.282–1.567)	p < 0.001
HABD	80/145 (55.17%)	125/158 (79.11%)	7/13 (53.84%)	1.434 (1.213–1.695)	p < 0.001
TID	35/67 (52.23%)	60/68 (88.23%)	0/1 (0%)	1.689 (1.322–2.158)	p < 0.001
CKD	57/92 (61.95%)	74/98 (75.51%)	4/6 (66.66%)	1.219 (1.002–1.482)	p < 0.001
GDs	47/85 (55.29%)	69/87 (79.31%)	1/2 (50.00%)	1.434 (1.152–1.786)	p < 0.001

Abbreviations – HABD: hydroelectrolytic and acid-base disorders; TID: tubulointerstitial diseases; CKD: chronic kidney disease; GDs: glomerular diseases; RR: Relative Risk; CI: confidence interval.

Regarding errors, in HABD, GPT-4 made 33 errors versus 65 for GPT-3.5, with 19 shared errors; 13 due to calculation errors in acid-base disorders, 5 in diagnosis, and 1 in management. In ITD, GPT-4 made 8 errors versus 32 for GPT-3.5, with 3 shared errors related to incorrect diagnoses. In CKD, GPT-4 made 24 errors, compared to 35 for GPT-3.5, with 2 shared in diagnosis. In GDs, GPT-4 made 18 errors and GPT-3.5 made 39, with 12 shared, 8 in management and 4 in diagnosis.

## Discussion

This is the first study analyzing AI performance in answering questions in nephrology tests in Brazil. GPT-4 was significantly better than GPT-3.5, achieving a 79.80% accuracy rate compared to 56.29% for GPT-3.5, a result supported by statistical tests including a chi-square test. GPT-4 showed superior results across all thematic areas, including hydroelectrolytic, acid-base, tubulointerstitial, chronic kidney, and glomerular diseases. Additionally, GPT-4 performed better on text-only questions and demonstrated fewer errors across all categories, highlighting its adaptability and better learning trajectory.

### General LLMs Studies

Accuracy studies involving GPT and other LLMs have been conducted across various fields. One study that included 861 questions from the Japanese National Medical Examination demonstrated an accuracy of 69.8% for GPT-4, compared to 41.9% for GPT-3.5 on multiple-choice questions^12^. Another study involving 1280 questions from four Taiwan Advanced Medical Licensing Examinations showed GPT-4’s accuracy on single-choice questions varied depending on the subject, with overall results ranging from 63.75% to 93.75%^13^. A third study, which examined 937 original multiple-choice questions from three written German medical licensing exams, reported superior performance by GPT-4 with 85%, significantly surpassing GPT-3.5’s score of 58%^14^. Similarly, an Israeli study evaluating only 3.5 on multiple-choice questions reported an accuracy of 36.6%, reinforcing the trend that GPT-4 generally outperforms GPT-3.5 in studies assessing LLMs’ performance on medical exam questions^15^.

### Nephrology LLMs Studies

In our nephrology-focused study, a consistent trend emerged, particularly highlighted by Wu et al.^16^ who exclusively examined 4 of six LLMs, finding it to be the most accurate with a rate of 73.3%. Similarly, Miao et al.^17^ demonstrated superior performance of version 4 with an accuracy of 74%, compared to 51% for version 3.5, further corroborating this trend. Additionally, Noda et al.^8^ contributed to these findings, albeit with marginally lower accuracies, yet still clearly demonstrating the superiority of version 4, which achieved an accuracy of 54.5% compared to 31.3% for 3.5.

Recent studies on ChatGPT’s performance in kidney disease exams demonstrate significant variations in accuracy and agreement between GPT-3.5 and GPT-4 across four major categories. Miao et al.18 evaluated GPT-3.5 on 150 questions concerning GDs, reporting an accuracy of 45% and 41% in two attempts. In contrast, Wu et al.^16^ showed that GPT-4 achieved an overall accuracy of 73.3% (629/858), with performance rates of approximately 70% in GDs, 80% in CKD, and 55% in HABD. Wu et al.^16^ also noted that fluid and acid-base disorders were the areas where the language model performed the worst. Noda et al.^8^ provided a more detailed comparison, indicating that GPT-4 outperformed GPT-3.5 in all categories: 43.5% accuracy in CKD with GPT-3.5, compared to 60.9% (14/23) with GPT-4. In GDs, GPT-3.5 achieved 28.6% (8/28), while GPT-4 scored 50.0% (14/28). For ITD, GPT-3.5 had 18.2% (2/11) accuracy, whereas GPT-4 reached 54.5% (6/11). In HABD, GPT-3.5 had a 40% accuracy (4/10), compared to 80% (8/10) for GPT-48, although the study presented a low number of questions. Specifically, our study found accuracies of 79.11% vs 56.29% in HABD, 88.23% vs 52.23% in ITD, 75.51% vs 61.95% in CKD, and 79.31% vs 55.29% in DG, showcasing substantial improvements over GPT-3.5 in these categories.

### Why is Nephrology a Challenge?

Nephrology is widely recognized as one of the most complex subspecialties within internal medicine^16^. In addition to this inherent complexity, it is well documented that large language models such as ChatGPT can produce “hallucinations”—outputs that are factually incorrect or irrelevant—thereby leading to controversial clinical reasoning and, at times, errors in mathematical calculations, including those required for acid-base disorder (HABD) formulas. Such errors have been found to be common across multiple GPT models^19^. Another significant concern is the reliability of the references used by LLMs to inform their clinical management and diagnostic recommendations; occasionally, these references may be unrealistic or even entirely fabricated^20,21^. To overcome these challenges, advanced techniques such as chain-of-thought prompting and retrieval-augmented generation (RAG) are necessary. These approaches help LLMs navigate the intricate reasoning required in nephrology by incorporating external, up-to-date information, thereby improving output reliability and reducing the incidence of hallucinations^22^.

### Questions with Images and Challenges

Focusing on studies that compared GPT-3.5 and GPT-4 for solving questions with and without images, Noda et al.^8^ reported that for 15 image-based questions, GPT-3.5 had an accuracy of 13.3%, while GPT-4 achieved 33.3%. The study also found a similar error rate for questions with and without images (20% and 23.8%, respectively), reinforcing GPT-4’s superiority over GPT-3.5 in answering questions8. Furthermore, it is important to highlight studies using GPT-4 to solve ECG-related questions. Gunay (2024) conducted a study in which 40 ECG questions without images were analyzed by GPT-4, with an overall accuracy of 36.33%^23^. In another study by Gunay (2024), the performance of GPT-4 and GPT-4o was evaluated on ECG questions with images, and their results were compared to those of cardiologists and the Gemini model. Across all ECG questions, cardiologists answered a median of 33.5 questions correctly (83.75%), GPT-4 answered a median of 20.5 (51.25%), GPT-4o answered 27, and Gemini answered 23 correctly^24^. Complementing these findings, our study achieved an overall accuracy of 81.49% for questions without images and only 54.54% for questions with images, with particular emphasis on electrocardiogram analysis, where we noted an accuracy of 60% in responses with a low number of only 10 questions.

Therefore, the lower performance in questions with images is due to the fact that GPT-4 and GPT-3 are primarily trained on textual data. Although GPT-4 has the inherent capability to process and interpret visual information—such as images, radiographs, or histological slides—when confronted with image-based questions, these models generally demonstrate inferior performance compared to dedicated multimodal models like GPT-4V and Gemini. Recent studies have shown that Med-Gemini, for example, outperformed both traditional GPT-4 and GPT-4V on multimodal medical benchmarks, such as the NEJM Image Challenges and the MMMU, with an average margin of 44.5% above GPT-4V^25^. Additionally, in some scenarios, the diagnostic accuracy of GPT-4V has approached that of human radiologists26.

### Gpt Versus Medical Students

Several studies have demonstrated that GPT-4 performs at or above the level of medical students on standardized medical and specialty licensing examinations. For example, the GPT-4 achieved an average accuracy of 85% on the German medical licensing examinations—ranking between the 92.8th and 99.5th percentile among students—and outperformed medical students and neurosurgery residents on neurosurgery questions, as well as outperforming Japanese medical residents on the General Medical Training Examination^14,27,28^. These findings suggest that GPT-4 has achieved a level of competence comparable to or above that of medical students across a range of countries and disciplines, highlighting its potential as a complementary tool in medical education and assessment.

### Open-Source vs. Proprietary LLMs in Medical Questions

GPT-4 generally outperforms open-source alternatives—including LLaMA, DeepSeek, and Med-PaLM—on a variety of medical benchmarks and clinical question sets. For instance, GPT-4 demonstrated higher accuracy than Meta’s LLaMA and Google’s Med-PaLM on several specialty exams, including otolaryngology, where GPT-4 achieved 77.1% accuracy compared to Med-PaLM’s 70.6% and LLaMA3:70b’s 66.8%^29^. Similarly, the open-source MEDITRON-70B model, while outperforming previous open models and even proprietary GPT-3.5, still remains slightly behind GPT-4 and Med-PaLM-2 on comprehensive medical benchmarks^30^. Despite this performance gap, open-source models are becoming increasingly viable alternatives, especially given that proprietary models like GPT-4 require paid licensing and may not always be accessible for all institutions or researchers. The open-source nature of models such as LLaMA and Med-PaLM allows for greater adaptability, transparency, and cost-effectiveness, supporting innovation and wider use in medical education and clinical research^31^.

### Ethical Aspects

We believe this study is important not only for GPT application in the learning processes of residents, with the potential to simulate clinical practice situations and enhance clinical reasoning, but also as a support for decision making. However, caution is warranted; despite ChatGPT adhering to the EU’s ethical AI guidelines, which emphasize human oversight, safety, privacy, transparency, diversity, societal impact, and accountability^32^, the content it generates may not always follow the most current and recommended guidelines^33^. That said, the implementation of GPT models as a tool in universities and medical residency emerges as an option for training and learning, but should never replace the physician’s role in the final decision making and conduct of patients.

### Limitations

This study provides insights into the capabilities of GPT models in answering questions in the nephrology residency exam but is constrained by several limitations. Notably, the limited inclusion of image-based questions (only 22 out of 411) restricts our ability to assess the models’ proficiency in visual data interpretation, which is essential for medical diagnostics, particularly in nephrology where imaging techniques such as ultrasound and CT scans are integral. Another important constraint was the inclusion of old questions, for example from 2010, which could bring up practices that are no longer recognized as first line in current nephrology practice, following the example of Rosol’s study, which used only recent evidence10. Furthermore, the scope of this study was confined to four specific themes within nephrology, omitting important nephrology topics like acute kidney injury and nephrolithiasis, which were analyzed by Wu et al.16. These areas are fundamental to a comprehensive understanding of nephrology and prevalent in clinical practice. Expanding the thematic coverage in subsequent studies will enhance the applicability of AI tools in nephrology, providing greater insights into their potential utility across the field’s diverse spectrum.

## Conclusion

This study demonstrated the superior performance of GPT-4 over GPT-3.5 in answering multiple-choice nephrology questions, with GPT-4 achieving a significantly higher accuracy across all thematic areas. These findings align with broader research, further confirming GPT-4’s enhanced capabilities, especially in text-based questions. However, the study’s limitations, including the small number of image-based questions and the absence of important nephrology topics, highlight the need for further study to better assess AI models’ full potential in clinical practice.

## Data Availability

The dataset supporting the findings of this study is not publicly available but can be obtained from the corresponding author upon reasonable request.
