# Supplementary material for: ChatGPT performance in answering medical residency questions in nephrology: a pilot study in Brazil
Source: J Bras Nefrol. 2025 Jul 4;47(4):e20240254. doi: 10.1590/2175-8239-JBN-2024-0254en (PMC12233955; doi:10.1590/2175-8239-JBN-2024-0254en)
Supplement: Translation

# Translation: Desempenho do ChatGPT na resposta a questões de residência médica em Nefrologia: um estudo piloto no Brasil

**DOI:** 10.1590/2175-8239-JBN-2024-0254pt

**Authors:** Helvécio Neves Feitosa, João Filipe Cavalcante Uchoa Furtado, Eduardo Correia Eulálio, Pedro Vianna Caldas Ribeiro, Lucas Macêdo Aurélio Paiva, Matheus Maia Gonçalves Bringel Correia, Geraldo Bezerra da Silva

**Affiliations:** 1 Universidade de Fortaleza, Faculdade de Medicina, Fortaleza, CE, Brasil.; 2 Universidade de Fortaleza, Faculdade de Medicina, Programas de Pós-Graduação em Saúde Pública e Ciências Médicas, Fortaleza, CE, Brasil.

**Keywords:** Inteligência Artificial Generativa, Modelos de Linguagem de Grande Escala, Exame de Residência Médica, Nefrologia

## Abstract

**Objetivo:**

Este estudo avaliou o desempenho das versões 4 e 3.5 do ChatGPT na resolução de questões de Nefrologia presentes em exames de residência médica no Brasil.

**Métodos:**

Foram analisadas 411 questões de múltipla escolha, com e sem imagens, organizadas em quatro temas principais: doença renal crônica (DRC), distúrbios do equilíbrio hidroeletrolítico e ácido-base (DHAB), doenças tubulointersticiais (DTI) e doenças glomerulares (DG). As questões com imagens foram respondidas somente pela versão ChatGPT-4. A análise estatística foi realizada utilizando o teste qui-quadrado.

**Resultados:**

O ChatGPT-4 alcançou uma precisão geral de 79,80%, enquanto o ChatGPT-3.5 obteve 56,29%, com uma diferença estatisticamente significativa (p < 0,001). Nos temas principais, o ChatGPT-4 teve desempenho superior em DHAB (79,11% vs. 55,17%), DTI (88,23% vs. 52,23%), DRC (75,51% vs. 61,95%) e DG (79,31% vs. 55,29%), todos com p < 0,001. O ChatGPT-4 apresentou uma acurácia de 81,49% nas questões sem imagens e de 54,54% em questões com imagens, com uma acurácia de 60% para análise de eletrocardiogramas. Este estudo é limitado pelo pequeno número de perguntas baseadas em imagens e pelo uso de itens de exame desatualizados, reduzindo sua capacidade de avaliar as habilidades diagnósticas visuais e a relevância clínica atual. Além disso, abordar somente quatro áreas da Nefrologia pode não representar totalmente a amplitude da prática nefrológica.

**Conclusão:**

Verificou-se que o ChatGPT-3.5 apresenta limitações no raciocínio sobre Nefrologia em comparação ao ChatGPT-4, evidenciando lacunas no conhecimento. O estudo sugere a necessidade de exploração adicional em outros temas da Nefrologia para aprimorar o uso dessas ferramentas de IA.

## Introdução

O ChatGPT é um aplicativo baseado em modelos de linguagem de grande escala (LLMs, do inglês *large language models*), como o GPT-4, desenvolvido pela OpenAI^1^. Esses modelos utilizam redes neurais complexas para processar e gerar linguagem natural de forma fluente e coerente^2^. A história dos LLMs tem início com os avanços no processamento de linguagem natural (PLN), com um aumento significativo na capacidade de gerar textos que se assemelham à escrita humana. Um exemplo desse avanço é o ChatGPT, que teve um grande impacto desde seu lançamento em 2022^3^. Seu treinamento envolve grandes volumes de dados e técnicas como ajuste fino e aprendizado por reforço a partir de feedback humano^4^. Essa inovação não apenas transformou as áreas de pesquisa, mas também revolucionou as interações com a inteligência artificial (IA) em diversas disciplinas^5^.

O ChatGPT tem sido amplamente utilizado para responder a perguntas complexas em exames de residência médica, demonstrando um desempenho promissor em diversas especialidades^6-8^. Por exemplo, em um estudo que avaliou sua capacidade de concluir o exame italiano de residência médica, o modelo obteve uma precisão geral de 90,44%, superando o desempenho de 99,6% dos participantes humanos^6^. Em outro estudo, o ChatGPT alcançou um desempenho semelhante ao de residentes em cirurgia plástica, respondendo corretamente a 55,8% das questões^7^. No campo da Nefrologia, o GPT foi testado em perguntas de autoavaliação, atingindo uma precisão de 54,5% com a versão GPT-4, que foi significativamente superior às versões anteriores, mas ainda abaixo do esperado para residentes experientes^8^.

O exame de residência médica no Brasil é uma prova obrigatória para graduados em medicina que buscam formação especializada. O exame é tradicionalmente completo, com questões de múltipla escolha e dissertativas, e é dividido em grandes áreas, como Cirurgia Geral, Pediatria, Obstetrícia e Ginecologia, Ciências Básicas, Saúde Pública e Clínica Médica^9^. Essa abordagem estruturada e abrangente garante que o conhecimento do candidato seja avaliado em uma variedade de áreas médicas, relevantes para a especialização desejada.

Compreender as diferenças de desempenho entre o GPT 3.5 e o 4 é fundamental, uma vez que cada iteração desses modelos traz melhorias que podem ser significativas em ambientes clínicos e educacionais. Por exemplo, o desempenho superior do GPT-4, conforme demonstrado no estudo de Rosoł et al.^10^, sugere uma maior aproximação do raciocínio humano em cenários médicos complexos, o que pode proporcionar uma ferramenta mais confiável para treinamento médico e suporte à tomada de decisões. Vale ressaltar que a importância dessa pesquisa vai além da curiosidade acadêmica. Ela se concentra no potencial de apoiar a educação médica por meio desses modelos, os quais funcionam como ferramentas avançadas de treinamento e avaliação, oferecendo simulações e auxílios de diagnóstico capazes de ajudar estudantes e profissionais da área médica a praticar e refinar seu raciocínio clínico em um ambiente livre de riscos. Além disso, à medida que os modelos de IA, como o GPT-4, continuam a evoluir, eles oferecem novas oportunidades para aprimorar o raciocínio clínico ao simular cenários de mundo real, permitir o acesso contínuo a conhecimentos médicos atualizados e facilitar o aprendizado individualizado por meio de feedback personalizado, contribuindo, em última análise, para uma educação médica mais adaptável e eficaz.

Neste estudo, comparamos o desempenho do GPT-4 e do GPT-3.5 na resposta a questões de múltipla escolha obtidas a partir de exames de residência em Nefrologia. Além disso, avaliamos os recursos dos modelos por meio da análise do percentual de acertos em diversos tópicos de Nefrologia. Para aprofundar a avaliação dos modelos de código aberto, realizamos uma análise comparativa dos erros comuns e fizemos avaliações de subgrupos com base no tipo de pergunta, incluindo aquelas com e sem componentes visuais.

## Métodos

### Gpts

Neste estudo, avaliamos a acurácia do GPT-4 e do GPT-3.5 na resposta a questões de múltipla escolha sobre Nefrologia. Foi empregada uma estratégia de acompanhamento de prompts, em que cada prompt incluiu “Contexto”, “Pergunta” e “Escolha” para potencializar a ampla capacidade de tokens de entrada do modelo, com um prompt por questão^11^. Caso o modelo não conseguisse fornecer uma resposta definitiva, a resposta era considerada incorreta. As avaliações foram realizadas utilizando uma GPU NVIDIA local para executar os comandos.

### Critérios de Inclusão

Foram selecionados somente exames oficiais de seleção para residência médica destinados a médicos generalistas e aplicados para admissão em programas de diversas subespecialidades, não exclusivamente em Nefrologia. As questões analisadas corresponderam à seção de Clínica Médica dessas provas, obtidas ativamente pelos pesquisadores em repositórios digitais públicos disponibilizados pelas próprias instituições de ensino. Não houve seleção aleatória: todas as questões relacionadas à Nefrologia, com e sem interpretação de imagens, presentes nesses exames oficiais foram incluídas para garantir a representatividade do conteúdo utilizado nos processos seletivos reais. É importante mencionar que as perguntas baseadas em imagens, incluindo aquelas sobre eletrocardiogramas, faziam parte das provas destinadas aos candidatos a médicos generalistas e não foram elaboradas especificamente para especialistas em Nefrologia. Cada questão apresentou um cenário clínico, seguido de um prompt para selecionar a única resposta correta dentre uma lista de opções. As perguntas não foram categorizadas por nível de dificuldade, já que a interpretação de tais exames pode variar bastante. O que é considerado desafiador para um candidato pode não ser para outro, dependendo de sua formação acadêmica e experiência anterior.

### Critérios de Exclusão

Foram excluídas as questões de Clínica Médica que não tinham relação com a Nefrologia. As perguntas que envolviam cenários de pacientes com tabelas complexas foram excluídas devido aos desafios que os LLMs têm para interpretar desfechos de pacientes apresentados em formato tabular. As 22 perguntas que exigiam interpretação de imagens foram incluídas apenas no GPT 4.0, uma vez que somente essa versão consegue ler imagens.

### Conjunto de Dados

Após o período de coleta de perguntas, nosso conjunto de dados foi composto por 411 questões de múltipla escolha extraídas de 310 exames de 161 instituições, divididas em quatro blocos principais: distúrbios do equilíbrio hidroeletrolítico e ácido-base (158 questões), doenças tubulointersticiais (68 questões), doença renal crônica (98 questões) e doenças glomerulares (87 questões). As perguntas foram selecionadas a partir de exames de residência médica realizados entre janeiro de 2010 e março de 2024, todos aplicados no idioma português do Brasil. As perguntas foram testadas em setembro de 2024. Das 411 questões, 389 sem imagens foram selecionadas para avaliação tanto pelo GPT-3.5 quanto pelo GPT-4.0, enquanto as 22 questões com imagens foram avaliadas exclusivamente pelo GPT-4.0. A Figura 1 resume o processo de captura e seleção de questões, bem como a criação do conjunto de dados.

**Figura 1:** Processo de coleta, seleção e análise das questões incluídas.

### Análise Experimental

Para a análise das questões, transferimos as informações para uma planilha do Microsoft Excel a fim de incorporar as respostas obtidas com o banco de testes. Como resultado, cada exemplo em nosso arquivo estruturado xlsx incluiu o ID da pergunta, o contexto, o prompt, as opções de resposta, a resposta correta e a área específica de Nefrologia à qual a questão pertencia. Utilizamos o *Statistical Package for the Social Sciences* (SPSS) versão 28.0 para realizar o teste qui-quadrado e comparar o desempenho dos dois modelos de GPT.

## Resultados

O GPT-4 apresentou um desempenho geral significativamente maior em comparação ao GPT-3.5, atingindo uma precisão de 79,80% (328/411) versus 56,29% (219/389) para o GPT-3.5, uma diferença que alcançou significância estatística (p < 0,001; RR: 1,418; IC 5%: 1,282–1,567). Quando o desempenho foi estratificado por subdomínios da Nefrologia, o GPT-4 superou consistentemente o GPT-3.5. Nos distúrbios do equilíbrio hidroeletrolítico e ácido-base (DHAB), o GPT-4 atingiu uma precisão de 79,11% (125/158) em comparação com 55,17% (80/145) do GPT-3.5 (RR: 1,434; IC 95%: 1,213-1,695; p < 0,001). Para doenças tubulointersticiais (DTI), as taxas foram de 88,23% (60/68) versus 52,23% (35/67) para GPT-4 e GPT-3.5, respectivamente (RR: 1,689; IC 95%: 1,322–2,158; p < 0,001). Na doença renal crônica (DRC), o GPT-4 atingiu 75,51% (74/98), enquanto o GPT-3.5 atingiu 61,95% (57/92), com um risco relativo de 1,219 (IC 95%: 1,002-1,482; p < 0,001). Nas doenças glomerulares (DG), o GPT-4 apresentou uma acurácia de 79,31% (69/87) em comparação com 55,29% (47/85) para o GPT-3.5 (RR: 1,434; IC 95%: 1,152–1,786; p < 0,001). Assim, em todos os domínios avaliados, o GPT-4 demonstrou uma precisão claramente superior e estatisticamente significativa em relação ao GPT-3.5.

O GPT-4 respondeu com sucesso 317 das 389 questões exclusivamente textuais e 12 das 22 questões com base em imagens, o que corresponde a 81,49% e 54,54%, respectivamente. A trajetória de respostas corretas em diversos bancos de questões indicou um aumento quase linear para o GPT-4, ao contrário do GPT-3.5, que não foi comparável em nenhuma avaliação segmentada (Figura 2). A Tabela 1 apresenta o resumo da quantidade de respostas corretas para LLMs de forma geral e nos tópicos.

**Figura 2:** Gráfico de linhas da precisão do GPT por área.

**Tabela 1:** Análise descritiva e comparativa do desempenho do GPT

Desempenho	GPT 3.5, n (%)	GPT 4.0, n (%)	Questões com imagem, n (%)	RR (IC95%); (GPT 4 vs. GPT 3.5)	Valor de p
Geral	219/389 (56,29%)	328/411 (79,80%)	12/22 (54,54%)	1,418 (1,282–1,567)	p < 0,001
DHAB	80/145 (55,17%)	125/158 (79,11%)	7/13 (53,84%)	1,434 (1,213–1,695)	p < 0,001
DTI	35/67 (52,23%)	60/68 (88,23%)	0/1 (0%)	1,689 (1,322–2,158)	p < 0,001
DRC	57/92 (61,95%)	74/98 (75,51%)	4/6 (66,66%)	1,219 (1,002–1,482)	p < 0,001
DG	47/85 (55,29%)	69/87 (79,31%)	1/2 (50,00%)	1,434 (1,152–1,786)	p < 0,001

Abreviações – DHAB: distúrbios do equilíbrio hidroeletrolítico e ácido-base; DTI: doenças tubulointersticiais; DRC: doença renal crônica; DG: doenças glomerulares; RR: Risco Relativo; IC: intervalo de confiança.

Com relação aos erros, nos DHAB, o GPT-4 cometeu 33 erros contra 65 do GPT-3.5, com 19 erros compartilhados; 13 devido a erros de cálculo em distúrbios ácido-base, 5 em diagnósticos e 1 em manejo. Em DTI, o GPT-4 cometeu 8 erros contra 32 do GPT-3.5, com 3 erros compartilhados relacionados a diagnósticos incorretos. Na DRC, o GPT-4 cometeu 24 erros, em comparação com 35 do GPT-3.5, com 2 erros compartilhados referentes a diagnósticos. Nas DG, o GPT-4 cometeu 18 erros e o GPT-3.5 cometeu 39, sendo 12 compartilhados, 8 no manejo e 4 no diagnóstico.

## Discussão

Este é o primeiro estudo a analisar o desempenho da IA na resolução de questões em exames de Nefrologia no Brasil. O GPT-4 foi significativamente melhor do que o GPT-3.5, alcançando uma taxa de precisão de 79,80% em comparação com 56,29% para o GPT-3.5, um resultado apoiado por testes estatísticos, incluindo o teste qui-quadrado. O GPT-4 demonstrou resultados superiores em todas as áreas temáticas, incluindo distúrbios hidroeletrolíticos, ácido-base, doenças tubulointersticiais, renais crônicas e glomerulares. Além disso, o GPT-4 teve um desempenho melhor em perguntas exclusivamente textuais e demonstrou menos erros em todas as categorias, destacando sua adaptabilidade e melhor trajetória de aprendizagem.

### Estudos Gerais Sobre LLMs

Estudos de precisão envolvendo o GPT e outros LLMs têm sido realizados em diversas áreas. Um estudo que incluiu 861 questões do Exame Nacional de Medicina do Japão demonstrou uma precisão de 69,8% para o GPT-4, em comparação com 41,9% para o GPT-3.5 em questões de múltipla escolha^12^. Outro estudo envolvendo 1.280 questões de quatro edições do Exame de Licenciamento Médico Avançado de Taiwan demonstrou que a acurácia do GPT-4 em questões de resposta única variou conforme o assunto, com resultados gerais entre 63,75% e 93,75%^13^. Um terceiro estudo, que analisou 937 questões de múltipla escolha originais provenientes de três provas escritas de exames de licenciamento médico na Alemanha, relatou um desempenho superior do GPT-4 com 85%, superando significativamente a pontuação do GPT-3.5 de 58%^14^. De forma semelhante, um estudo israelense que avaliou apenas a versão 3.5 em questões de múltipla escolha relatou uma precisão de 36,6%, reforçando a tendência de que o GPT-4 normalmente supera o GPT-3.5 em estudos que avaliam o desempenho de LLMs em questões de exames médicos^15^.

### Estudos Com LLMs em Nefrologia

Em nosso estudo focado em Nefrologia, surgiu uma tendência consistente, especialmente destacada por Wu et al.^16^ que analisaram exclusivamente a versão 4 dentre seis LLMs, considerando-a a mais precisa com uma taxa de 73,3%. Da mesma forma, Miao et al.^17^ demonstraram um desempenho superior da versão 4, com uma precisão de 74%, em comparação com 51% da versão 3.5, corroborando ainda mais essa tendência. Além disso, Noda et al.^8^ contribuíram para esses achados, embora com precisões marginalmente mais baixas, mas ainda assim demonstrando de forma clara a superioridade da versão 4, que alcançou uma acurácia de 54,5% em comparação com 31,3% da versão 3.5.

Estudos recentes sobre o desempenho do ChatGPT em exames de doenças renais demonstram variações significativas na precisão e na concordância entre as versões GPT-3.5 e GPT-4 em quatro categorias principais. Miao et al.^18^ avaliaram o GPT-3.5 em 150 perguntas sobre DG, relatando uma precisão de 45% e 41% em duas tentativas. Em contraste, Wu et al.^16^ demonstraram que o GPT-4 alcançou uma precisão geral de 73,3% (629/858), com taxas de desempenho de aproximadamente 70% em DG, 80% em DRC e 55% em DHAB. Wu et al.^16^ também observaram que os distúrbios de fluidos e ácido-base foram as áreas de pior desempenho do modelo de linguagem. Noda et al.^8^ forneceram uma comparação mais detalhada, indicando que o GPT-4 superou o GPT-3.5 em todas as categorias: 43,5% de precisão na DRC com o GPT-3.5, em comparação com 60,9% (14/23) com o GPT-4. Nas DG, o GPT-3.5 alcançou 28,6% (8/28), enquanto o GPT-4 obteve 50,0% (14/28). Para DTI, o GPT-3.5 teve 18,2% (2/11) de precisão, enquanto o GPT-4 alcançou 54,5% (6/11). Nos DHAB, o GPT-3.5 teve uma precisão de 40% (4/10), em comparação com 80% (8/10) para o GPT-4^8^, embora o estudo tenha apresentado um número reduzido de perguntas. Especificamente, nosso estudo encontrou precisões de 79,11% vs. 56,29% em DHAB, 88,23% vs. 52,23% em DTI, 75,51% vs. 61,95% em DRC e 79,31% vs. 55,29% em DG, demonstrando melhorias substanciais em relação ao GPT-3.5 nessas categorias.

### Por Que a Nefrologia é um Desafio?

A Nefrologia é amplamente reconhecida como uma das subespecialidades mais complexas da Clínica Médica^16^. Além dessa complexidade inerente, encontra-se bem documentado que os modelos de linguagem de grande escala, como o ChatGPT, podem produzir “alucinações” - dados que são factualmente incorretos ou irrelevantes - levando, assim, a um raciocínio clínico controverso e, às vezes, a erros em cálculos matemáticos, incluindo aqueles necessários para fórmulas de distúrbios ácido-base (DHAB). Tais erros têm se mostrado comuns em diversos modelos de GPT^19^. Outra preocupação significativa é a confiabilidade das referências utilizadas pelos LLMs para informar suas recomendações de manejo clínico e diagnóstico; ocasionalmente, essas referências podem ser irreais ou até mesmo totalmente fabricadas^20,21^. Para superar esses desafios, são necessárias técnicas avançadas, como o estímulo à cadeia de pensamento e a geração aumentada de recuperação (RAG, do inglês *retrieval-augmented generation*). Essas abordagens auxiliam os LLMs a navegar pelo intrincado raciocínio exigido em Nefrologia, incorporando informações externas e atualizadas, melhorando assim a confiabilidade dos dados e reduzindo a incidência de alucinações^22^.

### Questões Com Imagens e Desafios

Concentrando-se em estudos que compararam o GPT-3.5 e o GPT-4 na resolução de questões com e sem imagens, Noda et al.^8^ relataram que, para 15 questões baseadas em imagens, o GPT-3.5 demonstrou uma precisão de 13,3%, enquanto o GPT-4 alcançou 33,3%. O estudo também encontrou uma taxa de erro semelhante para perguntas com e sem imagens (20% e 23,8%, respectivamente), reforçando a superioridade do GPT-4 sobre o GPT-3.5 na resolução de perguntas^8^. Além disso, é importante destacar os estudos que utilizaram o GPT-4 para resolver questões relacionadas ao ECG. Gunay (2024) realizou um estudo no qual 40 questões sobre ECG sem imagens foram analisadas pelo GPT-4, com uma precisão geral de 36,33%^23^. Em outro estudo realizado por Gunay (2024), o desempenho do GPT-4 e do GPT-4o foi avaliado em questões de ECG com imagens, e seus resultados foram comparados aos dos cardiologistas e ao do modelo Gemini. Em todas as perguntas de ECG, os cardiologistas responderam corretamente a uma mediana de 33,5 perguntas (83,75%), o GPT-4 respondeu a uma mediana de 20,5 (51,25%), o GPT-4o respondeu a 27 e o Gemini respondeu corretamente a 23^24^. Complementando esses achados, nosso estudo alcançou uma precisão geral de 81,49% para questões sem imagens e apenas 54,54% para questões com imagens, com ênfase especial na análise de eletrocardiogramas, onde observamos uma precisão de 60% nas respostas com um número reduzido de apenas 10 questões.

Portanto, o desempenho inferior em perguntas com imagens se deve ao fato de o GPT-4 e o GPT-3 serem treinados principalmente em dados textuais. Embora o GPT-4 possua a capacidade inerente de processar e interpretar informações visuais - como imagens, radiografias ou lâminas histológicas - quando confrontado com perguntas baseadas em imagens, esses modelos geralmente demonstram desempenho inferior em comparação com modelos multimodais dedicados, como o GPT-4V e o Gemini. Estudos recentes demonstraram que o Med-Gemini, por exemplo, superou o desempenho tanto do GPT-4 tradicional quanto do GPT-4V em benchmarks médicos multimodais, como o *NEJM Image Challenges* e o MMMU, com uma margem média de 44,5% acima do GPT-4V^25^. Além disso, em alguns cenários, a precisão diagnóstica do GPT-4V se aproximou da de radiologistas humanos^26^.

### GPT Versus Estudantes de Medicina

Diversos estudos demonstraram que o GPT-4 apresenta desempenho igual ou superior ao de estudantes de Medicina em exames padronizados de licenciamento médico e de especialidades. Por exemplo, o GPT-4 alcançou uma precisão média de 85% nos exames de licenciamento médico da Alemanha - classificando-se entre os percentis 92,8 e 99,5 entre os estudantes - e superou o desempenho de estudantes de Medicina e residentes de Neurocirurgia em questões específicas da área, além de superar o desempenho de residentes de Medicina japoneses no Exame Geral de Treinamento Médico^14,27,28^. Esses resultados sugerem que o GPT-4 alcançou um nível de competência comparável ou superior ao de estudantes de medicina em diversos países e disciplinas, destacando seu potencial como ferramenta complementar na educação e avaliação médica.

### Llms de Código Aberto vs. Proprietários em Questões Médicas

O GPT-4 geralmente supera as alternativas de código aberto - incluindo LLaMA, DeepSeek e Med-PaLM - em uma variedade de referências médicas e conjuntos de questões clínicas. Por exemplo, o GPT-4 demonstrou acurácia superior ao LLaMA, da Meta, e ao Med-PaLM, do Google, em diversos exames especializados, incluindo otorrinolaringologia, onde o GPT-4 alcançou 77,1% de acurácia em comparação com 70,6% do Med-PaLM e 66,8% do LLaMA3:70b^29^. De maneira semelhante, o modelo de código aberto MEDITRON-70B, embora tenha superado modelos abertos anteriores e até mesmo o GPT-3.5 proprietário, ainda permanece um pouco atrás do GPT-4 e do Med-PaLM-2 em benchmarks médicos abrangentes^30^. Apesar dessa lacuna de desempenho, os modelos de código aberto estão se tornando alternativas cada vez mais viáveis, especialmente considerando que os modelos proprietários, como o GPT-4, exigem licenciamento pago e nem sempre podem ser acessíveis a todas as instituições ou pesquisadores. A natureza de código aberto de modelos como o LLaMA e o Med-PaLM permite maior adaptabilidade, transparência e custo-benefício, apoiando a inovação e o uso mais amplo na educação médica e na pesquisa clínica^31^.

### Aspectos Éticos

Acreditamos que este estudo é importante não apenas para a aplicação do GPT nos processos de aprendizagem de residentes, com o potencial de simular situações de prática clínica e aprimorar o raciocínio clínico, mas também como suporte para a tomada de decisões. No entanto, é necessário cautela; apesar de o ChatGPT aderir às diretrizes éticas da UE para IA, que enfatizam a supervisão humana, a segurança, a privacidade, a transparência, a diversidade, o impacto social e a responsabilidade^32^, o conteúdo gerado pode nem sempre seguir as diretrizes mais atuais e recomendadas^33^. Isso posto, a implementação de modelos de GPT como ferramenta em universidades e residências médicas surge como uma opção de treinamento e aprendizagem, mas jamais deve substituir o papel do médico na tomada de decisão final e na condução dos pacientes.

### Limitações

Este estudo fornece insights sobre as capacidades dos modelos de GPT em responder perguntas no exame de residência em Nefrologia, mas é restringido por diversas limitações. Em particular, a inclusão limitada de questões baseadas em imagens (apenas 22 de 411) restringe nossa capacidade de avaliar a proficiência dos modelos na interpretação de dados visuais, o que é essencial para diagnósticos médicos, especialmente em Nefrologia, onde técnicas de imagem, como ultrassom e TC, são essenciais. Outra limitação importante foi a inclusão de perguntas antigas, por exemplo, de 2010, que poderiam trazer à tona práticas que não são mais reconhecidas como de primeira linha na prática nefrológica atual, seguindo o exemplo do estudo de Rosol, que utilizou apenas evidências recentes^10^. Além disso, o escopo deste estudo foi limitado a quatro temas específicos dentro da Nefrologia, omitindo tópicos importantes da área, como injúria renal aguda e nefrolitíase, que foram analisados pelo estudo de Wu et al.^16^. Essas áreas são fundamentais para uma compreensão abrangente da Nefrologia e são prevalentes na prática clínica. A ampliação da cobertura temática em estudos subsequentes aumentará a aplicabilidade das ferramentas de IA em Nefrologia, fornecendo maiores insights sobre sua utilidade potencial em todo o espectro diversificado da especialidade.

## Conclusão

Este estudo demonstrou o desempenho superior do GPT-4 em relação ao GPT-3.5 na resposta a questões de múltipla escolha em Nefrologia, com o GPT-4 obtendo uma precisão significativamente maior em todas as áreas temáticas. Esses achados estão em consonância com pesquisas mais amplas, confirmando ainda mais os recursos aprimorados do GPT-4, especialmente em questões baseadas em texto. No entanto, as limitações do estudo, incluindo o pequeno número de perguntas baseadas em imagens e a ausência de tópicos importantes de Nefrologia, destacam a necessidade de estudos adicionais para uma avaliação mais precisa de todo o potencial dos modelos de IA na prática clínica.

## Data Availability

O conjunto de dados que apoia os achados deste estudo não está disponível publicamente, mas podem ser obtidos mediante solicitação ao autor correspondente.
